# Nanocluster-Based Ultralow-Temperature Driven Oxide Gate Dielectrics for High-Performance Organic Electronic Devices

**DOI:** 10.3390/ma13235571

**Published:** 2020-12-07

**Authors:** Jeong-Wan Jo, Jingu Kang, Kyung-Tae Kim, Seung-Han Kang, Jae-Cheol Shin, Seung Beom Shin, Yong-Hoon Kim, Sung Kyu Park

**Affiliations:** 1 Department of Electrical Engineering, University of Cambridge, Cambridge CB2 1TN, UK; jj531@cam.ac.uk; 2 School of Electrical and Electronic Engineering, Chung-Ang University, Seoul 06974, Korea; flykkokko@cau.ac.kr (J.K.); rudxo4812@cau.ac.kr (K.-T.K.); seunghank@cau.ac.kr (S.-H.K.); tlswo0627@cau.ac.kr (J.-C.S.); kyocu6007@cau.ac.kr (S.B.S.); 3 School of Advanced Materials Science and Engineering, Sungkyunkwan University, Suwon 16419, Korea; 4 SKKU Advanced Institute of Nanotechnology (SAINT), Sungkyunkwan University, Suwon 16419, Korea

**Keywords:** solution-processed metal-oxide gate dielectrics, deep ultraviolet (DUV) photochemical activation, low-temperature process, organic thin-film transistor, single-crystal organic semiconductor

## Abstract

The development of novel dielectric materials with reliable dielectric properties and low-temperature processibility is crucial to manufacturing flexible and high-performance organic thin-film transistors (OTFTs) for next-generation roll-to-roll organic electronics. Here, we investigate the solution-based fabrication of high-k aluminum oxide (Al_2_O_3_) thin films for high-performance OTFTs. Nanocluster-based Al_2_O_3_ films fabricated by highly energetic photochemical activation, which allows low-temperature processing, are compared to the conventional nitrate-based Al_2_O_3_ films. A wide array of spectroscopic and surface analyses show that ultralow-temperature photochemical activation (<60 °C) induces the decomposition of chemical impurities and causes the densification of the metal-oxide film, resulting in a highly dense high-k Al_2_O_3_ dielectric layer from Al-13 nanocluster-based solutions. The fabricated nanocluster-based Al_2_O_3_ films exhibit a low leakage current density (<10^−7^ A/cm^2^) at 2 MV/cm and high dielectric breakdown strength (>6 MV/cm). Using this dielectric layer, precisely aligned microrod-shaped 2,7-dioctyl[1]benzothieno [3,2-b][1] benzothiophene (C8-BTBT) single-crystal OTFTs were fabricated via solvent vapor annealing and photochemical patterning of the sacrificial layer.

## 1. Introduction

Solution-processable organic thin-film transistors (OTFTs) are attractive building blocks for application in low-cost, large-area, and flexible/wearable electronics, owing to their flexibility, light weight, low process temperature, and solution processability [1,2,3,4,5,6,7,8,9]. Single-crystal organic semiconductors offer superior electrical performance and stability compared to polycrystalline or amorphous organic semiconductors. Hence, considerable efforts have been made in improving the single-crystal growth methodology and charge transport to enhance the performance of solution-processed OTFTs [10,11,12,13,14,15,16,17]. For the industrial implementation of solution-processed organic electronics, the development of a reliable gate dielectric is essential because the electrical performance of OTFTs strongly depends on the quality of the gate dielectric. To achieve high-performance OTFTs, the gate dielectrics must exhibit a frequency-independent high dielectric constant for low-voltage operation, low gate leakage current, high breakdown voltage, and favorable interface with the organic semiconductors. Furthermore, low-temperature processing and solution processability are also important for simple and low-cost flexible/wearable electronics. To meet these requirements, a variety of polymer gate dielectrics have been explored with the advantages of solution processability, low process temperature, and good compatibility with the organic semiconductor [18,19,20,21,22]. However, the use of polymer dielectrics in high-performance organic electronics is limited, owing to their relatively poor stability, low dielectric constant, and weak chemical and physical endurance compared to inorganic counterparts.

Low-temperature solution-processed high-k metal-oxide dielectrics have been proposed as promising alternative candidates for emerging flexible/wearable electronics [23,24]. Many novel chemical approaches and post-treatment methodologies have been investigated to develop low-temperature solution-processed high-k oxide dielectrics, including combustion fuel additives [25,26,27], carbon-free water solvents [28,29,30,31], inorganic-organic hybrid [32,33,34,35], and ultraviolet (UV) activation [36,37,38,39,40,41]. However, these engineered precursor systems and innovative processing strategies either impose restrictions on the gate electrode selection or require relatively high process temperatures (~300 °C), limiting the usage of low thermal budget polymer substrates. In particular, in the case of room-temperature UV activation, an extremely thin oxide dielectric film is achieved because of the limited penetration depth of the UV light and relatively slow chemical reaction of the conventional sol–gel precursor [38,42]. These conditions demand careful preparation of the dielectric layer (because even a small number of defects can cause significant failure such as high gate leakage current in these thin dielectric layers) and thereby hinder the facile application for the development of relatively high voltage driven OTFTs. Additionally, solution-based single-layer metal-oxide gate dielectric films without any additive layers, fabricated by room-temperature UV activation, typically exhibit slightly lower electrical performance, possibly due to the loosely bonded amorphous network, low film density, and defects both in the dielectric bulk as well as at the interface with the semiconductor layers [34,35]. These drawbacks need to be alleviated for realizing high-performance organic electronics while retaining a low thermal budget manufacturing advantage. Therefore, it is strongly required to investigate a novel metal precursor optimized for solution-processed high-k oxide dielectric utilizing low-temperature UV photochemical activation.

In this communication, we propose a novel strategy to fabricate ultralow-temperature (<60 °C) solution-processed aluminum oxide (Al_2_O_3_) dielectric films for high-performance solution-based OTFTs. By employing [Al_13_(μ_3_-OH)_6_(μ-OH)_18_(H_2_O)_24_]-(NO_3_)_15_ (Al-13 nanoclusters) and conventional aluminum nitrate with local structure controllable photoactivation process, we systematically investigate the structural properties of the dielectric films and characterize the electrical properties with the implementation of parallel capacitors and single-crystal OTFTs. The microstructure and chemical composition of the solution-processed Al_2_O_3_ via photochemical activation as well as the dielectric properties using both aluminum precursors are compared in detail. The 2,7-dioctyl [1]benzothieno [3,2-*b*][1]benzothiophene (C8-BTBT) organic single-crystal microrods are successfully grown on nanocluster-based Al_2_O_3_ dielectrics via photochemical activation and solvent vapor annealing (SVA). We demonstrate that the nanocluster-based Al_2_O_3_ dielectrics enable the solution-based fabrication of OTFTs with a high-performance (>28 cm^2^ V^−1^ s^−1^) and relatively low operation voltage (<−20 V) using precisely aligned C8-BTBT single crystal as the semiconductor. The results reported here imply that the nanocluster-based oxide dielectric materials offer a general route to high-performance, extremely stable, solution-based OTFTs with minimal heat input, facilitating compatibility with standard complementary metal-oxide-semiconductor (CMOS) processing and scalable on-chip device applications.

## 2. Materials and Methods

Solutions for nitrate- and nanocluster-Al_2_O_3_ were prepared by dissolving 0.78 M aluminum nitrate nonahydrate (Al(NO_3_)_3_·9(H_2_O), Sigma-Aldrich, St. Louis, MO, USA) and 0.06 M Al-13 nanocluster precursors in 2-methoxyethanol (2-ME) (anhydrous, Sigma-Aldrich, St. Louis, MO, USA), respectively. When the precursors were dissolved in the solvent, the solutions were thoroughly sonicated for more than 2 h. For Al_2_O_3_ film deposition, the solutions were spin-coated at 3000 rpm for 20 s onto a heavily doped p-type silicon wafer, and deep ultraviolet (DUV) annealing was performed at 60 °C for 2 h under nitrogen flow. Then, polymethylmethacrylate (PMMA, A4 950, Microchem Corp., Newton, MA, USA) was spin-coated on SiO_2_/Si or Al_2_O_3_/Si substrate as a polymeric sacrificial layer at 2000 rpm for 40 s and baked at 150 °C for 30 min. For the photochemical patterning of the PMMA layer, DUV irradiation was performed under continuous nitrogen flow for 30 min using a chrome-patterned quartz photomask. A 0.5 wt % of C8-BTBT solution prepared by dissolving the powder in chlorobenzene was spun onto the pre-patterned PMMA at a spin rate of 3000 rpm for 20 s. To form a single-crystal C8-BTBT microrod, SVA was performed for 15 h in a chloroform-filled glass container. Finally, 10 nm of MoO_3_ and 60 nm of Au were thermally evaporated as an efficient charge injection layer and source/drain electrodes using a shadow mask to fabricate the OTFTs. The crystalline states of C8-BTBT polycrystalline films and microrod-shaped single crystals were characterized by cross-polarized optical microscopy (CPOM, LV100ND POL/DS, Nikon Instruments Korea, Seoul, Korea). The X-ray diffraction (XRD) spectra were obtained using D8-Advance (Bruker-AXS, Karlsruhe, Germany) and analyzed by the EVA software (9.0 version, Bruker-AXS, Karlsruhe, Germany). Fourier transform infrared (FT-IR) spectroscopy (Nicolet 6700, Thermo Scientific, Waltham, MA, USA) was used under nitrogen purging condition. X-ray photoemission spectroscopy (XPS) images were captured using K-alpha + (Thermo Fisher Scientific, Waltham, MA, USA) with an Al Kα source at 1486 eV and a base pressure of 7.8 × 10^−9^ mbar. The thickness and atomic force microscopy (AFM) images of films were measured using ellipsometry (SpecEL-2000) and XE-100 AFM system (Park Systems Corp., Suwon, Korea), respectively. The capacitance versus frequency (C-F) and leakage current density versus electric field (J-E) measurements were performed on top metal (IZO)-insulator (Al_2_O_3_)-bottom metal (heavily doped p-type silicon) (MIM) structures with an overlapped area of 100 × 100 μm^2^. The dielectric constants were extracted from the MIM capacitance values measured at 100 Hz. The capacitance and leakage current properties were measured by using an Agilent LCR meter 4284A and an Agilent 4156C analyzer, respectively. The electrical characterization of the C8-BTBT OTFTs was performed using an Agilent 4156C semiconductor parameter analyzer under dark and ambient conditions.

## 3. Results and Discussion

**Figure 1:**
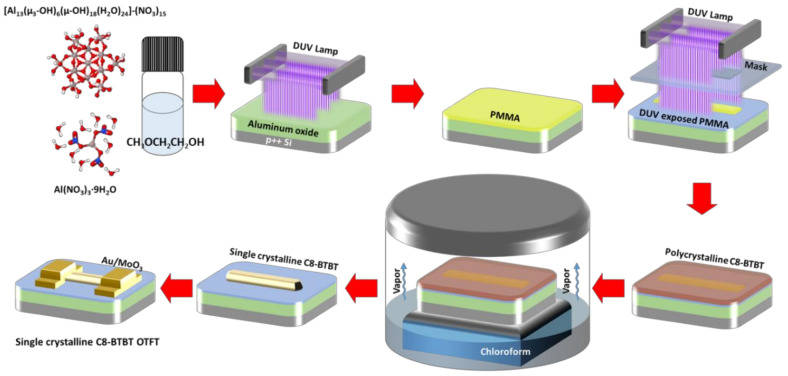
Schematic illustration of the fabrication of single-crystal C8-BTBT organic thin-film transistors (OTFTs) based on solution-processed Al_2_O_3_ gate dielectric films.

Figure 1 shows the schematic fabrication of single-crystal C8-BTBT OTFTs based on solution-processed Al_2_O_3_ gate dielectric films. To fabricate Al_2_O_3_ dielectric films, a precursor solution containing conventional aluminum nitrate or Al-13 nanocluster was spin-coated on the heavily doped p-type silicon wafer and subsequently annealed via room-temperature DUV (wavelengths of 254 and 185 nm) irradiation. Then, the PMMA layer was spin-coated on Al_2_O_3_ dielectric layers as a sacrificial layer to form the organic single crystals at specific positions and control their growth directions. High energetic photons irradiated from DUV lamp through a pre-patterned photomask spatially modified the PMMA layer. Then, the polycrystalline C8-BTBT film was fabricated on the photochemically modified PMMA layer by spin-coating. After the SVA in a chloroform-filled glass container for up to 12 h, a high aspect ratio C8-BTBT single-crystalline formed preferably on the pristine PMMA regions. Finally, molybdenum oxide (MoO_3_) and Au were deposited on the C8-BTBT single crystal as source/drain electrodes.

**Figure 2:**
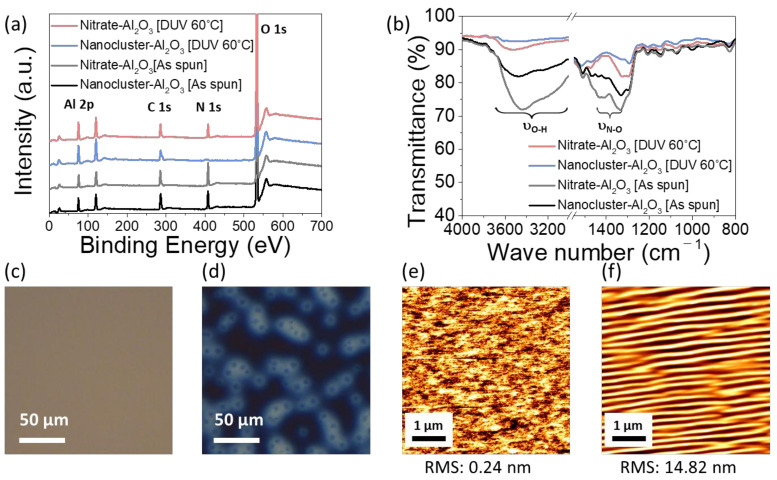
(**a**) XPS survey scan and (**b**) FT-IR spectra showing the compositional change of nanocluster- and nitrated-based Al_2_O_3_ film with deep ultraviolet (DUV) annealing at 60 °C. Optical microscope (OM) images of (**c**) nanocluster-based Al_2_O_3_ film and (**d**) nitrate-based Al_2_O_3_ film. Atomic force microscopy (AFM) noncontact-mode topography of (**e**) nanocluster-based Al_2_O_3_ and (**f**) nitrate-based Al_2_O_3_ film.

The versatility of this approach, such as its potential in nanocluster-based low-temperature photoactivated dielectric materials, is demonstrated by a variety of spectroscopic analyses and electrical characterizations. X-ray photoelectron spectroscopy (XPS) was utilized to characterize the compositional changes in the film deposited using aluminum nitrate and Al-13 nanocluster solution upon DUV irradiation (Figure 2a). Surface etching by Ar sputtering was not performed to prevent the compositional change; thus, carbon peaks were observed on the surfaces of all samples. In the as-spun films, carbon and nitrogen were detected, owing to the precursor and solvent, respectively. After DUV annealing at 60 °C for 120 min, the nanocluster-based Al_2_O_3_ film did not contain any nitrogen, whereas the nitrogen peak was retained in the nitrate-based Al_2_O_3_ film. This indicates that the nitrate-based Al_2_O_3_ film forms incomplete metal-oxide-metal (M-O-M) networks despite UV irradiation. As shown in Figure 2b, the FT-IR spectra indicate that the intensities of the broad IR absorbance centered at ~3500 cm^−1^ (O-H stretching) in DUV-annealed nanocluster-based Al_2_O_3_ film significantly decreased [43,44]. Another important feature in the FT-IR spectra of the DUV-annealed nanocluster-based Al_2_O_3_ film is the disappearance of absorption bands centered at 1400 and 1340 cm^−1^ (N-O stretching) [45]. These spectroscopy analyses distinctly indicate the effective formation of a highly dense Al_2_O_3_ film using an Al-13 nanocluster precursor under ultralow-temperature (60 °C) DUV photochemical activation. Figure 2c,d show the optical micrographs of the surfaces of photoactivated nanocluster- and nitrate-based Al_2_O_3_ films exposed to the atmosphere for more than 12 h. The uniformity of the nanocluster precursor film is retained excellently over time. However, when the nitrate-based Al_2_O_3_ film was exposed to the atmosphere, a circular defect formed, which was different from the initial stage. The uniformity of the nitrate-based Al_2_O_3_ film appears to be damaged by moisture, owing to the insufficient removal of residual solvent molecules and chemical impurities that interfere in the formation of the M-O-M network [46]. AFM images indicated that, after irradiation, the surface of the nanocluster-based Al_2_O_3_ was smooth and uniform (Figure 2e), showing the root-mean-square roughness of the film, 0.22 nm, which is comparable to thermally grown commercial SiO_2_ [47]. For the nitrate-based Al_2_O_3_ films (DUV-annealed at 60 °C), a very rough surface morphology of 14.82 nm was observed (Figure 2f), which is consistent with the above analysis of the incomplete structural formation of metal-oxide films (or M-O-M networks). The wave pattern observed in the AFM image is considered as the swelling of the metal-oxide thin film due to moisture absorption in the loose M-O-M networks.

**Figure 3:**
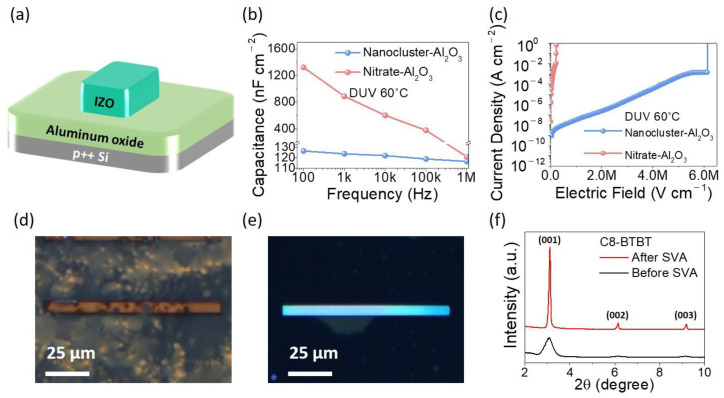
(**a**) Schematic illustration of IZO/Al2O3/p++ Si capacitor structure. (**b**) Leakage current density as a function of the electric field (J-E) and (**c**) capacitance–frequency (C-F) characteristics of nanocluster- and nitrate-based Al_2_O_3_ gate dielectrics. cross-polarized optical microscopy (CPOM) images of (**d**) polycrystalline C8-BTBT film spun on patterned polymethylmethacrylate (PMMA) layer and (**e**) 100 μm long C8-BTBT microrod-shaped single crystal preferably located at the pristine PMMA region after solvent vapor annealing (SVA) for 15 h. (**f**) XRD spectra of the as-spun film (before SVA) and microrod single-crystal arrays (after SVA) of C8-BTBT.

The dielectric properties of the nanocluster- and nitrate-based Al_2_O_3_ films were assessed by fabricating an IZO/Al_2_O_3_/p++ Si capacitor structure (Figure 3a). Figure 3b,c show the capacitance versus frequency (C-F) and current density versus electric field (J-E) characteristics of nanocluster- and nitrate-based Al_2_O_3_ dielectric films. The capacitances of nanocluster- and nitrate-based Al_2_O_3_ films were 887 and 123 nF/cm^2^, respectively, at 1 kHz. Despite the ultralow process temperature, the nanocluster-Al_2_O_3_ film (DUV-annealed at 60 °C) showed a sufficiently densified thickness of 56.7 nm and a relatively high ε_r_ of 7.93, whereas the nitrate-based Al_2_O_3_ film showed a thickness of 239.1 nm and ε_r_ of 357, possibly due to insufficient condensation and densification of the films. While the nanocluster-based Al_2_O_3_ film exhibited a stable frequency dependence, as shown in Figure 3b, the nitrate-based Al_2_O_3_ film exhibited an abnormally high capacitance value at low frequency and a highly unstable frequency dependence owing to a large number of M-OH bonds caused by the incompletely formed M-O-M network. The nanocluster-based Al_2_O_3_ film exhibited a lower leakage current density (3.5 × 10^−8^ A cm^−2^) at 1 MV cm^−1^ compared to nitrate-based Al_2_O_3_ films (breakdown before 1 MV cm^−1^), as shown in Figure 3c. The nanocluster-based Al_2_O_3_ film also demonstrated a sufficiently high breakdown field (>6 MV cm^−1^). The electrical characteristics of the nanocluster-based Al_2_O_3_ exceed those of the nitrate-based Al_2_O_3_, which is attributed to the formation of highly dense M-O-M networks of Al_2_O_3_ film with fewer impurities and minimal structural defects, owing to the optimally designed nanocluster precursors for high-energy photoactivation. Furthermore, to confirm the stability of the nanocluster-based Al_2_O_3_ dielectric films over time, we conducted time-dependent dielectric breakdown (TDDB) experiments at different constant voltage stress conditions, as shown in Figure S1 and Table S1. From the TDDB analysis, using a *E*^1/2^ model at a 63.2% failure rate, it was found that the nanocluster-based Al_2_O_3_ can achieve a 10-year lifetime when operated at a maximum electric field of 1.20 MV/cm.

To evaluate the crystallinity of the C8-BTBT single-crystal microrod on the nanocluster-based Al_2_O_3_ film via photochemical activation and SVA, we analyzed CPOM images, as shown in Figure 3d,e. In general, it is noted that the bright color of the organic semiconductor region in the CPOM image corresponds to high crystallinity [16]. Figure 3d demonstrates that the as-spun C8-BTBT films on a photochemically modified PMMA layer exhibited the polycrystalline nature of C8-BTBT small grains. High aspect ratio single-crystal C8-BTBT was observed on the pristine PMMA regions after the SVA in chloroform, as shown in Figure 3e. Furthermore, we performed XRD to evaluate the crystallinity of as-spun C8-BTBT films and the microrod C8-BTBT single crystal (Figure 3f). In both cases, three peaks are observed at the same degrees and assigned to the (00l) reflections [48,49,50]. The significantly narrow and high peaks of the microrod C8-BTBT single crystal in the XRD spectrum suggest that the C8-BTBT single crystal with a high degree of crystallinity was successfully grown on nanocluster-based Al_2_O_3_ films [50].

**Figure 4:**
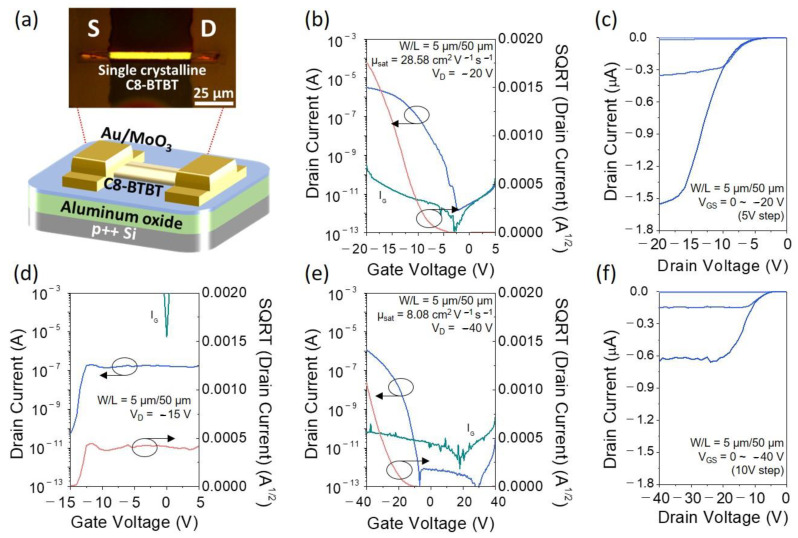
(**a**) Schematic and OM image of C8-BTBT single-crystal TFT structure on Si substrate. Transfer and output characteristics of C8-BTBT OTFT using (**b**,**c**) nanocluster-based Al_2_O_3_, (**d**) nitrate-based Al_2_O_3_, and (**e**,**f**) conventional SiO_2_.

To ensure the viability of the developed ultralow-temperature photoactivated nanocluster systems, the single-crystal C8-BTBT OTFT was implemented using a nanocluster-based Al_2_O_3_ gate dielectric. Figure 4a shows the schematic diagram of the device structure and OM image of the single-crystal C8-BTBT OTFTs, and Figure 4b,c exhibit typical transfer and output characteristics of the single-crystal C8-BTBT OTFTs. To calculate field-effect mobility, we used the 11.98 and 29.74 nF/cm^2^ as the serial capacitance of PMMA/SiO_2_ and PMMA/Al_2_O_3_ bilayer dielectric, respectively. The single-crystal C8-BTBT OTFTs exhibit p-type behaviors, with maximum saturation mobility of >28.58 cm^2^/(V·s) and on/off current ratio of >2.1 × 10^6^ with the subthreshold swing of 1.27 V/dec. (Figure 4b). On the other hand, C8-BTBT OTFTs fabricated with the nitrate-based Al_2_O_3_ demonstrate almost no functional electrical characteristics, possibly due to the high gate leakage currents, as shown in Figure 4d. For comparison, we fabricated single-crystal C8-BTBT OTFTs on conventional 200 nm SiO_2_ using the same processing conditions. The corresponding electrical characteristics are shown in Figure 4e,f. The field-effect hole mobility, on/off ratio, and subthreshold swing estimated from Figure 4e are 8.08 cm^2^/(V·s), 7.2 × 10^6^, and 0.78 V/dec. respectively. Despite applying an ultralow thermal budget (DUV-annealed at 60 °C), the C8-BTBT OTFTs with nanocluster-based Al_2_O_3_ dielectrics have shown relatively superior electrical properties compared to the C8-BTBT OTFTs with SiO_2_ dielectrics, which is attributed to minimal interfacial trap states with organic semiconductors and less bulk defects in the nanocluster-based Al_2_O_3_ gate dielectrics. These results indicate that the ultralow-temperature fabricated nanocluster-based Al_2_O_3_ film includes sufficient dielectric properties and chemical durability for the implementation of gate dielectric materials in high-performance organic semiconductor TFT applications. We also fabricated and compared the various TFTs using poly-crystalline C8-BTBT, carbon nanotube (CNT), and inorganic indium oxide (In_2_O_3_) to demonstrate the general applicability of the nanocluster-based Al_2_O_3_ gate dielectric films. As shown in Figure S2, the poly-crystalline C8-BTBT OTFT and CNT TFT exhibit p-type behaviors, with maximum saturation mobility of 0.07 cm^2^/(V·s) and 0.53 cm^2^/(V·s), respectively. In addition, the inorganic In_2_O_3_ TFT exhibits n-type behaviors with maximum saturation mobility of 5.31 cm^2^/(V·s). These results demonstrate that ultralow-temperature photoactivated nanocluster-based Al_2_O_3_ gate dielectrics are generally applicable to various charge transport materials.

## 4. Conclusions

In this research, we explored a novel strategy for ultralow-temperature solution-processed Al_2_O_3_ dielectric materials and demonstrated the implementation of high-performance OTFTs to ensure the viability of the developed materials. Two solution-processable aluminum precursors, namely the nanocluster and conventional nitrate, were compared for the low-temperature Al_2_O_3_ gate dielectrics. XPS, FT-IR, and AFM studies showed that a highly energetic photochemical activation can be an effective method to form highly dense nanocluster-based Al_2_O_3_ thin films by the efficient dissociation and rearrangement of the Al-13 nanocluster precursor. Furthermore, the single-crystal C8-BTBT microrod was precisely grown at the desired locations by the spatial photochemical modification of polymeric sacrificial layers and subsequent SVA. With nanocluster-based Al_2_O_3_ gate dielectric, single-crystal C8-BTBT OTFTs exhibited a high on-off current ratio of >2.1 × 10^6^, subthreshold swing of <1.27 V/decade, and field-effect mobility of >28.58 cm^2^/(V·s). Together with these results for the utilization of high-performance gate dielectric materials that can be fabricated at ultralow process temperatures (~60 °C), the nanocluster-based Al_2_O_3_ films can be attractive candidates as solution-processable gate dielectrics for roll-to-roll processed high-performance organic electronics.

## Supplementary Materials

The following are available online at https://www.mdpi.com/1996-1944/13/23/5571/s1, Figure S1: Time-dependent dielectric breakdown (TDDB) experiments at different constant voltage stress conditions of the nanocluster-based Al_2_O_3_, Figure S2: Representative transfer characteristics of poly-crystalline C8-BTBT, CNT, and indium oxide TFTs based on the nanocluster-based Al_2_O_3_., Table S1: Summary of the extrapolated maximum operation voltages of the nanocluster-based Al_2_O_3_ dielectric films comparing the lifetime models for 63.2, 10, and 1 % failure rate for 10 years lifetime.
