# Supplementary material for: Nanocluster-Based Ultralow-Temperature Driven Oxide Gate Dielectrics for High-Performance Organic Electronic Devices
Source: Materials (Basel). 2020 Dec 7;13(23):5571. doi: 10.3390/ma13235571 (PMC7730230; doi:10.3390/ma13235571)
Supplement: Supplementary file 1 [file materials-13-05571-s001.pdf]

materials

Supplementary Materials

Nanocluster-Based Ultralow-Temperature Driven Oxide Gate Dielectrics for High-Performance Organic Electronic Devices

Jeong-Wan Jo [1], Jingu Kang [2], Kyung-Tae Kim [2], Seung-Han Kang [2], Jae-Cheol Shin [2], Seung Beom Shin [2], Yong-Hoon Kim [3,4,*] and Sung Kyu Park [2,*]

[1] Department of Electrical Engineering, University of Cambridge, Cambridge CB2 1TN, UK; jj531@cam.ac.uk
[2] School of Electrical and Electronic Engineering, Chung-Ang University, Seoul 06974, Korea; flykkokko@cau.ac.kr (J.K.); rudxo4812@cau.ac.kr (K.-T.K.); seunghank@cau.ac.kr (S.-H.K.); tlswo0627@cau.ac.kr (J.-C.S.); kyocu6007@cau.ac.kr (S.B.S.)
[3] School of Advanced Materials Science and Engineering, Sungkyunkwan University, Suwon 16419, Korea
[4] SKKU Advanced Institute of Nanotechnology (SAINT), Sungkyunkwan University, Suwon 16419, Korea
* Correspondence: yhkim76@skku.edu (Y.-H.K.); skpark@cau.ac.kr (S.K.P.)

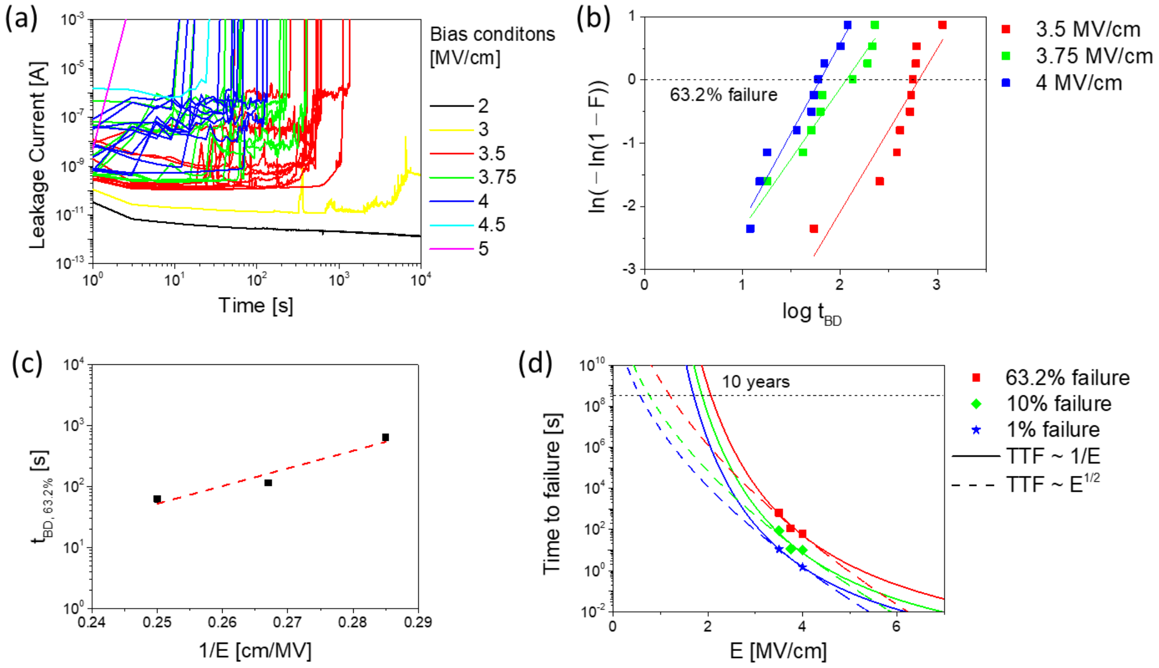

Figure S1. (**a**) Gate leakage current over time during a constant voltage stress for selected bias conditions of 2–5 MV/cm. (**b**)Weibull plot for the bias-dependent TDDB measurements conducted on nanocluster-based Al_2O_3 dielectric films. (**c**) Weibull scaling factor (t_{BD} at 63.2% failure) from bias-dependent TDDB measurements as a function of 1/*E*. (**d**) Lifetime of the nanocluster-based Al_2O_3 gate dielectric at lower gate voltages extrapolated from TDDB experiments. TTF for 63.2, 10, and 1% failure rate is shown. 1/*E* model (solid line) and $E^{1/2}$ model (dotted line) were used to extrapolate the maximum operation voltage.

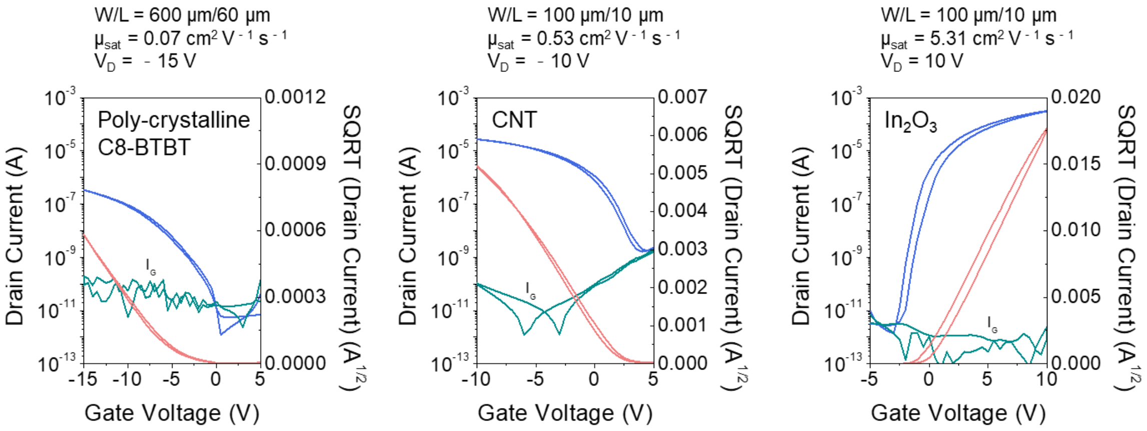

Figure S2. Representative transfer characteristics of poly-crystalline C8-BTBT, CNT, and indium oxide TFTs based on the nanocluster-based Al_2O_3.

Table S1. Summary of the extrapolated maximum operation voltages of the nanocluster-based Al_2O_3 dielectric films comparing the lifetime models for 63.2, 10, and 1 % failure rate for 10 years lifetime.

Model (Failure Rate)	Maximum Operation Electric Field
1/E (63.2%)	2.07 MV/cm
1/E (10%)	1.88 MV/cm
1/E (1%)	1.69 MV/cm
$E^{1/2}$ (63.2%)	1.20 MV/cm
$E^{1/2}$ (10%)	0.75 MV/cm
$E^{1/2}$ (1%)	0.56 MV/cm

© 2020 by the authors. Licensee MDPI, Basel, Switzerland. This article is an open access article distributed under the terms and conditions of the Creative Commons Attribution (CC BY) license (http://creativecommons.org/licenses/by/4.0/).
